# Adverse birth outcomes among mothers who received intermittent preventive treatment with Sulphadoxine-Pyrimethamine in the low malaria transmission region

**DOI:** 10.1186/s12884-019-2397-1

**Published:** 2019-07-08

**Authors:** Wigilya P. Mikomangwa, Minzi OMS, Eleni Aklillu, Appolinary A. R. Kamuhabwa

**Affiliations:** 1 Clinical Pharmacy and Pharmacology Department, Muhimbili University of Health and Allied Sciences, Dar es Salaam, Tanzania; 2 Division of Clinical Pharmacology, Department of Laboratory Medicine, Karolinska Institutet, Karolinska University Hospital-Huddinge C1:68, SE-141 86 Stockholm, Sweden

**Keywords:** Maternal anemia, Fetal anemia, Preterm delivery, LBW and IPT-p-SP

## Abstract

**Background:**

Malaria in pregnancy increases the risk of adverse birth outcomes such as low birth weight (LBW), maternal and foetal anemia. In Tanzania, some areas have attained low malaria transmission. However, data on the burden of preterm delivery, LBW, maternal and foetal anemia following substantial reduction of malaria transmission in recent years is still scarce in these settings.

**Methods:**

A study involving 631 pregnant women was conducted at Mwananyamala referral hospital in Dar es Salaam from April to August, 2018. Study enrollment was done prior to delivery. Structured interview and antenatal clinic cards were used to obtain data including the use of intermittent preventive therapy in pregnancy using sulfadoxine-pyrimethamine (IPTp-SP). Infants birth weights were recorded, maternal venous and cord blood were taken for testing of malaria and determination of haemoglobin (Hb) levels. Chi-square test and regression analysis were done to identify risk factors for preterm delivery, LBW, maternal and foetal anemia.

**Results:**

The prevalence of malaria among mothers who used at least one dose of IPTp-SP was 0.6% (4/631). Fourteen mothers (2.2%) did not use IPTp-SP and had no malaria infection. The prevalence of maternal anemia, LBW, foetal anemia and preterm delivery was 40.6, 6.5, 5.9 and 9.2% respectively. Participants who were malaria positive had 11 times more risk of LBW compared to those who were negative (AOR, 11; 95%, CI 1.07–132.2; *p* = 0.04). The risk of delivering babies with LBW was 1.12 times high among mothers who were ≤ 36 weeks of gestation (AOR, 1.12; 95% CI, 0.06–0.25; *p* = < 0.001). The use of ≥3 doses of IPTp-SP was associated with 83% decrease in risk of LBW compared to those who did not use any dose of IPTp-SP (AOR, 0.17; 95% CI, 0.03–0.88; *p* = 0.05). Severe anaemia at delivery was associated with seven times increased risk of preterm delivery compared to non-anemic participants (AOR, 6.5; 95% CI, 1.49–28.16; *p* = 0.013).

**Conclusion:**

Despite the reduced malaria transmission and use of IPTp-SP, prevalence of preterm delivery, maternal anemia, LBW and foetal anemia is still high in Tanzania. The recommended ≥3 doses of IPTp-SP should continue be provided even in areas with substantial reduction of malaria.

## Background

Adverse birth outcomes such as maternal anemia, LBW, fetal anemia and preterm delivery contribute to maternal and newborn morbidity worldwide [1–3]. In Sub-Saharan Africa (SSA) adverse birth outcomes are augmented by malaria endemicity [3]. Maternal anemia is an independent predictor of other adverse birth outcomes such as fetal anemia, preterm delivery, small for gestation age, intrauterine growth restriction and, LBW [4]. Around 800 million children and women are affected by anemia [4]. The global prevalence of anemia among women of reproductive age and pregnant women is approximately 29.4 and 38.2%, respectively. The prevalence of anemia among pregnant women is higher in Africa (44.6%) followed by Asia (39.3%). In Tanzania, the prevalence of anemia and severe anemia among women of reproductive age is 40% and 2%, respectively [4, 5]. The regions with a high prevalence of anemia also have a high prevalence of factors that contribute to anemia, such as malaria, sickle cell disease and thalassemia [6]. Other causes of anemia include worm infestation, schistosomiasis, HIV infection, inadequate bioavailable of dietary iron, folic acid and vitamin B_12_ [6, 7].

As a result of these, WHO recommends a package of preventive measures against worm infestations, malaria and iron deficiency [8]. At every visit to the antenatal clinic (ANC), pregnant women should receive a dose of monthly Sulphadoxine-Pyrimethamine (1500mg/75mg) for intermittent preventive treatment (IPTp-SP) starting early in the second trimester to delivery. Besides, one dose of Mebendazole (500mg) is given at every ANC visit after first trimester. Iron/folic acid supplements (60 mg (mg) of iron and 400 micrograms (μg/d) of folic acid) should be taken daily from the first trimester until delivery. Pregnant women are also advised to sleep under insecticide-treated nets (ITN) and use indoor residual spray (IRS) during the whole period of pregnancy [3]. It has been reported by various studies that, anemia in pregnancy increases the risk of adverse birth outcomes to both the woman and newborn. These adverse birth outcomes include fetal anemia, LBW, preterm birth, intrauterine growth restriction and perinatal mortality [1, 9, 10]. Therefore, it was expected that, effective implementation of WHO recommendations for the prevention of malaria and anemia would reduce maternal adverse birth outcomes particularly in SSA in which the burden is significant. For instance, preterm delivery is more prevalent (85%) in Africa and Asia than in developed countries such as Europe (3.6%) [11]. In Tanzania, the incidence of preterm delivery is approximately 12% [11].

In SSA where malaria transmission is high, malaria significantly causes maternal anemia, fetal anemia, LBW and preterm delivery and contributes to the complications during pregnancy. In these regions, controlling malaria can reduce anemia by more than a quarter and 60% of severe anemia [6]. Tanzania, as one of the countries in SSA, has a high prevalence of malaria (7.3%) with some regions having substantially reduced malaria prevalence to less than 1% [12]. The reduced malaria transmission has also been associated with a reduced prevalence of LBW in Rwanda [13]. Data on the prevalence of LBW, maternal, fetal anemia and preterm delivery in areas with reduced malaria transmission in Tanzania is scarce. Information on the burden of adverse birth outcomes is important to institute the required measures for improvement of maternal and child health in these regions. Therefore, the objective of this study was to determine the prevalence of maternal anemia, LBW, fetal anemia and preterm delivery in a region with a low prevalence of malaria among pregnant women aged ≥18 years (1.1%).

## Methods

### Study design and study area

The study design was a facility-based observational cross-sectional study to identify the prevalence of adverse birth outcomes such as LBW, maternal anemia, fetal anemia, and preterm delivery as well as the associated risk factors. The study was conducted at Mwananyamala referral hospital situated in Kinondoni Municipality in Dar es Salaam. The prevalence of malaria in Dar-es-Salaam has declined from 6.4% in 2002 to 1.1% in 2017. On average, about 30 pregnant women deliver at Mwananyamala hospital per day, making an average of 850 deliveries per month. The hospital has a dedicated clinic, which provides antennal care including the provision of IPTp-SP and other WHO recommended preventive methods for malaria and anemia during pregnancy.

### Study population

Pregnant women admitted in labor wards to deliver during April to August 2018 were recruited and enrolled into the study. The eligibility criteria were pregnant women aged 18 years or above and residing in the study areas for at least 6 months before enrolment in the study. Also, pregnant women who delivered singleton vaginally, known HIV negative as of the last visit to the ANC, were included in the study. Pregnant women with a complicated pregnancy and other co-morbidities such as pre-eclampsia, eclampsia, hemorrhages (> 50 ml blood loss), sepsis and chronic diseases were excluded from the study. Pregnant women whose ANC cards or medical forms were incomplete were excluded.

### Sample size

The sample size was calculated using single population proportion formula considering 95% confidence interval (CI) and proportion of 50% (15) with 4% margin of error and 5% non-respondent rate as follows; *n* = Z_α/2_P (1-P)/ε^2^ where n is sample size, ε is the marginal error. Finally, 631 pregnant women admitted in labor wards before delivery were consecutively enrolled.

### Data collection

A total of 790 pregnant women were screened during recruitment process using a validated screening tool. One hundred and fifty nine (159) of the screened pregnant women were excluded from the study; 4 pregnant women were HIV positive, 1 had incomplete records (incomplete antenatal clinic card), 2 had complicated pregnancy and were referred to National Hospital, and 152 hemorrhaged. Antenatal clinic cards were used to collect data on HIV status, IPTp-SP, mebendazole and iron/folic acid (FEFO) use. Participants were interviewed to verify the information about the use of FEFO, IPTp-SP, and mebendazole during pregnancy. After delivery maternal venous blood and cord blood was taken within 30 and 5 min respectively using EDTA containing blood collection tubes for laboratory analysis. Infants were weighed within 30 min after delivery using baby weighing scale.

The study outcomes for this study were the prevalence of adverse birth outcomes including maternal anemia, LBW, fetal anemia and preterm delivery. Sociodemographic and obstetric characteristics data were collected using a structured case record form. Socio-demographic characteristics included age, education level, marital status, attendance to ANC, use of Iron/folic acid supplements. The use of IPTp-SP, mebendazole, and insecticide-treated nets, and indoor residual spray was documented. The assessed obstetric characteristics included gravidity and gestation age. Gestation age was determined by last normal menstrual period.

### Laboratory analysis of blood samples

Maternal and fetal Hb levels were determined using HemoCue® Hb 201^+^ HemoCue AB, Angelholm, Sweden. About 5 μL of blood was used for testing malaria infection using malaria Rapid diagnostic test, (mRDT), SD BIOLINE Malaria Ag P.f/pan, Standard Diagnostics, INC.

#### Case definitions

Case definitions of study outcomes were according to WHO definitions: Maternal Hb (anemia in pregnancy) was categorized as normal, mild, moderate and severe anemia when the Hb concentrations were ≥ 11.0 g/dL, 10.0–10.9 g/dL, 7–9.9 g/dl, and ≤ 7 g/dL, respectively. Fetal Hb was characterized as normal when ≥12.5 g/dL or anemic when < 12.5 g/dL. Birth weight was defined as low when the baby weighed < 2.5Kg and normal when ≥2.5Kg. Preterm delivery was considered when a pregnant woman delivered before 37 weeks of gestation [9, 15].

### Data analysis

Mean and median with standard deviation were used to summarize continuous variables. A chi-square test or Fisher exact test was used to compare categorical variables, row and column percentages were used when appropriate. Univariate analysis was used to determine the factors associated with preterm delivery, LBW, maternal and fetal anemia. Variables with *p* < 0.2 were subjected to multivariate analysis. Crude odds ratios (OR), adjusted odds ratios (AOR) at 95% CI with *p*-values < 0.05 were regarded to be statistically significant. Data were analyzed using a Statistical Package for Social Sciences (SPSS) program version 20.0.

## Results

### Characteristics of study participants

A total of 631 pregnant women aged 18–45 years with a mean of 26.27 ± 5.42 years old participated in the study. The majority (44.1%) were aged 18–24 years. About three quarters (75.1%) of them were married and 58.0% had attained primary education. Concerning gravidity, 38.5, 27.4, and 34.1% were primigravida, secundigravida, and multigravida, respectively. The majority (90.8%) of participants were at ≥37 weeks of gestation and most (65.0%) of them had visited antenatal clinics at least four times during their recent pregnancies (Table 1).

**Table 1:** Socio-demographic, Obstetric and clinical characteristics of study participants and association with maternal anemia

Characteristics	Number of women n (%)	Maternal anemia		OR (95% CI)	*p*-value	AOR(95% CI)	*P*-value
		Yes n (%)	No n (%)				
Age groups							
18–24	278 (44.1)	113 (44.1)	165 (44.0)	1.2 (0.68–2.10)	0.54		
25–29	177 (28.1)	70 (27.3)	107 (28.5)	1.3 (0.69–2.26)	0.46		
30–34	116 (18.4)	46 (18.0)	70 (18.7)	1.2 (0.66–2.34)	0.50		
> 34	60 (9.5)	27 (10.5)	33 (8.8)	1			
Marital status							
Married	474 (75.1)	66 (25.8)	91 (24.3)	0.9 (0.64–1.33)	0.67		
Unmarried	157 (24.9	190 (74.2)	284 (75.7)	1			
Education level							
No formal education	25 (4.0)	13 (5.1)	12 (3.2)	0.5 (0.16–1.82)	0.32		
Primary education	366 (58.0)	149 (58.2)	217 (57.9)	0.9 (0.33–2.21)	0.74		
Secondary education	221 (35.0)	87 (34.0)	134 (35.7)	0.9 (0.34–2.37)	0.83		
Tertiary education	19 (3.0)	7 (2.7)	12 (3.2)	1			
Attendance to ANC							
< 4	221 (35.0)	93 (36.3)	128 (34.1)	0.9 (0.65–1.27)	0.57		
≥ 4	410 (65.0)	163 (63.7)	247 (65.9)	1			
FEFO use							
Yes	613 (97.1)	6 (2.3)	12 (3.2)	1.4 (0.51–3.72)	0.53		
No	18 (2.9)	250 (97.7)	363 (96.8)	1			
Mebendazole use							
Yes	596 (94.5)	20 (7.8)	15 (4.0)	1			
No	35 (5.5)	236 (92.2)	360 (96.0)	2.0 (1.02–4.05)	0.04	1.9 (0.95–3.90)	0.07
Gravidity							
Primigravida	243 (38.5)	99 (38.7)	144 (38.4)	1.2 (0.81–1.70)	0.40	1.22 (0.84–1.78)	0.30
Secundigravida	173 (27.4)	61 (23.8)	112 (29.9)	1.5 (0.98–2.24)	0.06	1.51 (0.997–2.30)	0.052
Multigravida	215 (34.1)	96 (37.5)	119 (31.7)	1			
Gestation age (weeks)							
≤ 36	58 (9.2)	25 (9.8)	33 (8.8)	1.1 (0.65–1.94)	0.68		
≥ 37	573 (90.8)	231 (90.2)	342 (91.2)	1			
Number of IPTp-SP doses							
0	14 (2.2)	8 (3.1)	6 (1.6)	0.5 (0.16–1.36)	0.16	1.17 (0.36–3.77)	0.80
1	70 (11.1)	35 (13.7)	35 (9.3)	0.6 (0.37–1.03)	0.06	1.72 (0.56–5.24)	0.34
2	169 (26.8)	69 (27.0)	100 (26.7)	0.9 (0.62–1.29)	0.54	1.99 (0.67–5.94)	0.22
≥ 3	378 (59.9)	144 (56.2)	234 (62.4)	1			
Malaria by mRDT							
Positive	4 (0.6)	3 (1.2)	1 (0.3)	4.4 (0.46–42.87)	0.198	3.6 (0.344–37.70)	0.29
Negative	627 (99.4)	253 (98.8)	374 (99.7)	1			
ITN use							
Yes	619 (98.1)	251 (98.0)	368 (98.1)	1.05 (0.33–3.34)	0.938		
No	12 (1.9)	5 (2.0)	7 (1.9)	1			
IRS use							
Yes	394 (37.6)	159 (62.1)	235 (62.7)	1.01 (0.74–1.42)	0.89		
No	237 (62.4)	97 (37.9)	140 (37.3)	1			

Adjusted for mebendazole use, gravidity, IPTp-Sp use and malaria

Mebendazole (94.5%) and FEFO (97.1%) were used by the majority of pregnant women. The median use of IPTp-SP was three doses, while 14 (2.2%) participants did not use any dose of IPTp-SP during the current pregnancy. On the other hand, 11.1, 26.8, 39.6 and 20.3% of pregnant women used 1, 2, 3 and ≥ 4 doses of IPTp-SP, respectively. Of the 631 study participants, 4 (0.6%) were malaria positive at the time of delivery (Table 1). Among those who were malaria positive, one reported to have used a single dose of IPTp-SP and others used three doses of IPTp-SP.

### Prevalence of maternal anemia and associated risk factors

The mean Hb concentration at delivery was 11.21 ± 1.62 g/dL. The prevalence of severe, moderate and mild anemia was 1.4, 16.3, and 23%, respectively, while the overall prevalence of anemia at delivery was 40.6%. On univariate analysis, mebendazole use was associated with two times more protection against anemia compared to not using mebendazole (OR, 2.0 95% CI 1.02–4.05, *p* = 0.04). After adjusting for covariates, the use of mebendazole did not affect maternal anemia (*p* = 0.07). The use of IPTp-SP, gravidity, gestation age at delivery, marital status, level of education, number of visits to the ANC and age of pregnant women were not associated with maternal anemia at delivery (Table 1).

### Prevalence of LBW and associated risk factors

The mean birth weight among 631 babies was 3.1 ± 0.46Kg. The prevalence of LBW (< 2.5Kg) was 6.5%, including 16 (2.5%) babies born preterm and 25 (4.0%) born full term. The risk of delivering LBW babies was higher among mothers at < 37 weeks of gestation than those at ≥37 weeks of gestation (AOR, 1.12; 95% CI, 0.06–0.25), *p* = < 0.01). Participants who were aged 25–29 years had 80% reduced risk of LBW compared to those aged > 35 years (AOR, 0.20; 95%CI, 0.05–0.84; *p* = 0.03). On univariate analysis, the prevalence of LBW among delivered babies was significantly higher in primigravida (9.9%) and multigravida (5.1%) than secundigravida (3.5%) mothers (*p* = 0.02). There was no statistical significant on the prevalence of LBW among primigravida and multigravida, (*p* = 0.43). On multivariate analysis, primigravida was not associated with LBW (*p* = 0.08). The risk of LBW was not modified by the level of maternal anemia (mild, moderate and severe anemia) among participants with low malaria prevalence (0.6%). On multivariate analysis, participants who had malaria infection had 12 times increased risk of LBW compared to those who had no malaria at delivery (AOR, 11.9; 95% CI, 1.07–132.2; *p* = 0.04). Moreover, participants who used ≥3 doses of IPTp-SP had 83% reduced risk of LBW (AOR, 0.17; 95% CI, 0.03–0.88; *p* = 0.04). Marital status, level of education, age of participants, ITN, IRS and FEFO use, number of visits to the antenatal clinics were not associated with LBW (Table 2).

**Table 2:** Association between LBW and Sociodemographic, obstetric and clinical characteristics of 631 pregnant women

Variable	LBW		OR (95% CI)	*p*-value	AOR (95% CI)	*p*-value
	No n (%)	Yes n (%)				
Age groups						
18–24	254 (92.0)	22 (8.0)	0.8 (0.30–2.0)	0.60	0.25 (0.06–1.04)	0.06
25–29	171 (95.5)	8 (4.5)	0.4 (0.14–1.28)	0.13	0.20 (0.05–0.84)	*0.03*
30–34	110 (95.7)	5 (4.3)	0.4 (0.12–1.39)	0.15	0.32 (0.09–1.19)	0.09
≥ 35	55 (90.2)	6 (9.8)	1			
Marital status						
Unmarried	148 (94.3)	9 (5.7)	1.2 (0.56–2.55)	0.65		
Married	442 (93.2)	32 (6.8)				
Education level						
No formal education	24 (96.0)	1 (4.0)	0.8 (0.04–12.82)	0.84		
Primary education	342 (93.4)	24 (6.6)	1.3 (0.16–9.87)	0.82		
Secondary education	206 (93.2)	15 (6.8)	1.3 (0.16–10.50)	0.80		
Tertiary education	18 (94.7)	1 (5.3)	1			
Attendance to ANC						
< 4	206 (91.2)	20 (8.8)	1.7 (0.88–3.14)	0.12	1.33 (0.62–2.89)	0.46
≥ 4	384 (94.8)	21 (5.2)	1			
FEFO use						
No	17 (94.4)	1 (5.6)	0.8 (0.11–6.49)	0.87		
Yes	573 (93.5)	40 (6.5)	1			
Mebendazole use						
No	34 (97.1)	1 (2.9)	0.4 (0.06–3.06)	0.38		
Yes	556 (93.3)	40 (6.7)	1			
Gravidity						
Primigravida	219 (90.1)	24 (9.9)	3.1 (1.22–7.63)	0.02	3.0 (0.88–10.04)	0.08
Multigravida	204 (94.9)	11 (5.1)	1.5 (0.544–4.143)	0.43	1.07 (0.30–3.85)	0.92
Secundigravida	167 (96.5)	6 (3.5)	1			
Gestation age (weeks)						
≤ 36	42 (72.4)	16 (27.6)	8.4 (4.14–16.88)	< 0.01	1.12 (0.06–0.25)	*< 0.01*
≥ 37	548 (95.6)	25 (4.4)	1			
IPTp-SP doses taken						
0	12 (85.7)	2 (14.3)	1			
1	64 (91.4)	6 (8.6)	0.56 (0.10–3.31)	0.51	0.25 (0.04–1.56)	0.13
2	155 (91.7)	14 (8.3)	0.54 (0.11–2.67)	0.45	0.32 (0.06–1.74)	0.19
≥ 3	359 (95.0)	19 (5.0)	0.32 (0.07–1.52)	0.15	0.17 (0.03–0.88)	*0.04*
Malaria by MRDT						
Positive	3 (75)	1 (25)	4.5 (0.50–48.1)	0.17	11.9 (1.07–132.2)	*0.04*
Negative	587 (93.6)	40 (6.4)	1			
Maternal anemia						
Non-anemia (≥11.0 g/dL	349 (93.3)	25 (6.7)	1			
Mild anemia (10–10.9 g/dL)	139 (95.9)	6 (4.1)	0.242–1.501	0.28	1.16 (0.11–12.6)	0.9
Moderate anemia (7.0–9.9 g/dL)	94 (91.3)	9 (8.7)	0.603–2.960	0.48	0.51 (0.04–5.78)	0.58
Severe anemia (< 7.0 g/dL)	8 (88.9)	1 (11.1)	0.21–14.510	0.61	1.01 (0.10–9.99)	0.995
Foetal anemia						
Yes	34 (91.9)	3 (8.1)	0.78 (0.23–2.64)	0.68		
No	556 (93.6)	38 (6.4)	1			

Adjusted for age, ANC, gravidity, gestation age, IPTp-Sp use, malaria infection and maternal anemia

### Prevalence of preterm delivery and associated risk factors

Gestation age at delivery ranged from 28 to 45 weeks with a mean gestation age of 38.8 ± 1.77 weeks. The prevalence of preterm delivery was 9.2%. Primigravida was at two times increased risk of preterm delivery compared to multigravida (AOR, 1.97; 95% CI, 1.01–3.81; *p* = 0.045). The use of IRS was not associated significantly with preterm delivery (*p* = 0.06) on univariate analysis, after adjusting for covariates on multivariate analysis, the use of IRS was associated with preterm delivery (AOR, 0.50; 95% CI, 0.27–0.95; *p* = 0.03). Participants who had severe anemia at delivery were at seven times increased risk of preterm delivery compared to non-anemia participants (AOR, 6.5; 95% CI, 1.49–28.16; *p* = 0.013) (Table 3).

**Table 3:** Association between fetal anemia, preterm delivery, and Sociodemographic, obstetric and clinical characteristics of 631 pregnant women

	Foetal Anemia		OR (95% CI)	*p*-value	Preterm delivery					
	Yes 37 (%)	No 594 (%)			Yes	No	OR (95%CI)	*p*-value	AOR (95%CI)	*p*-value
Variable										
Age groups										
18–24	12 (32.4)	266 (44.8)	0.5 (0.17–1.47)	0.21	31 (11.2)	245 (88.8)	1.4 (0.53–3.81)	0.49		
25–29	10 (27.0)	167 (28.1)	0.7 (0.22–2.01)	0.46	13 (7.3)	166 (92.7)	0.88 (0.30–2.57)	0.81		
30–34	10 (27.0)	106 (17.8)	1.0 (0.34–3.19)	0.95	9 (7.8)	106 (92.2)	0.95 (0.30–2.97)	0.93		
> 34	5 (13.5)	55 (9.3)	1		5 (8.2)	56 (91.8)	1			
Marital status										
Unmarried	6 (16.2)	151 (25.4)	0.6 (0.23–1.39)	0.21	11 (7.0)	146 (93.0)	0.68 (0.35–1.36)	0.28		
Married	31 (83.8)	443 (74.6)	1		47 (9.9)	427 (90.1)	1			
Education level										
No formal education	3 (8.1)	22 (3.7)	2.5 (0.24–25.67)	0.45	0 (0.0)	25 (100)	0.0	1		
Primary education	25 (67.6)	341 (57.4)	1.3 (0.17–10.29)	0.80	32 (8.7)	334 (91.3)	0.51 (0.14–1.85)	0.31		
Secondary education	8 (21.6)	213 (35.9)	0.7 (0.08–5.71)	0.72	23 (10.4)	198 (89.6)	0.62 (0.17–2.29)	0.47		
Tertiary education	1 (2.7)	18 (3.0)	1		3 (15.8)	16 (84.2)	1			
Attendance to ANC										
< 4	11 (29.7)	210 (35.4)	0.8 (0.38–1.60)	0.49	24 (10.6)	202 (89.4)	1.3 (0.75–2.25)	0.36		
≥ 4	26 (70.3)	384 (64.6)	1		34 (8.4)	371 (91.6)	1			
FEFO use										
No	3 (8.1)	15 (2.5)	3.4 (0.94–12.34)	0.06	0 (0.0)	18 (100.0)	0.0 (0.0)	1		
Yes	34 (91.9)	579 (97.5)	1		58 (9.5)	555 (90.5)	1			
Mebendazole use										
No	2 (5.4)	33 (5.6)	1.0 (0.22–4.22)	0.97	3 (8.6)	32 (91.4)	0.92 (0.27–3.11)	0.90		
Yes	35 (94.6)	561 (94.4)	1		55 (9.2)	541 (90.8)	1			
Gravidity										
Primigravida	12 (32.4)	231 (38.9)	0.7 (0.34–1.65)	0.5	31 (12.8)	212 (87.2)	1.95 (1.02–3.72)	*0.043*	1.97 (1.01–3.81)	*0.045*
Secundigravida	11 (29.7)	162 (27.3)	1.0 (0.43–2.21)	1.0	12 (6.9)	161 (93.1)	0.99 (0.45–2.18)	0.99	0.95 (0.43–2.13)	0.91
Multigravida	14 (37.8)	201 (33.8)	1		15 (7.0)	200 (93.0)	1		1	
IPTp-SP doses taken										
0	0 (0.0)	14 (2.4)	0	1.0	0 (0.0)	14 (100.0)	0.0 (0.0)	1	0.0 (0.0)	1
1	6 (16.2)	64 (10.8)	1.7 (0.65–4.34)	0.29	11 (15.7)	59 (84.3)	1.89 (0.91–3.93)	0.09	1.87 (0.87–4.02)	0.11
2	11 (29.7)	158 (26.6)	1.2 (0.58–2.66)	0.57	13 (7.7)	156 (92.3)	0.84 (0.43–1.64)	0.62	0.83 (0.42–1.63)	0.58
≥ 3	20 (54.1)	358 (60.3)	1		34 (9.0)	344 (91.0)	1		1	
Malaria by MRDT										
Positive	0 (0.0)	4 (0.7)	–	1.0	0 (0.0)	4 (100.0)	0.0 (0.0)	1		
Negative	37 (100.0)	590 (99.3)	–		58 (9.3)	569 (90.7)	1			
ITN use										
Yes	37 (100)	582 (98.0)	1		55 (8.9)	564 (91.1)	1		1	
No	0 (0.0)	12 (2.0)	0.0 (0.0)	1	3 (25.0)	9 (75.0)	3.4 (0.90–13.0)	0.07	3.31 (0.84–13.03)	0.09
IRS use										
Yes	21 (56.8)	373 (62.8)	1		43 (10.9)	351 (89.1)	1		1	
No	16 (43.2)	221 (37.2)	1.29 (0.66–2.52)	0.46	15 (6.3)	222 (93.7)	0.55 (0.30–1.02)	0.06	0.50 (0.27–0.95)	*0.03*
Maternal anemia										
Non-anemia (≥11.0 g/dL)	18 (48.6)	356 (59.9)	1		3 (33.3)	6 (66.7)	1		1	
Mild anemia (10–10.9 g/dL)	12 (32.4)	133 (22.4)	1.8 (0.837–3.805)	0.13	11 (10.7)	92 (89.3)	0.85 (0.42–1.73)	0.65	0.78 (0.38–1.62)	0.50
Moderate anemia (7.0–9.9 g/dL)	6 (16.2)	97 (16.3)	1.2 (0.473–3.166)	0.68	11 (7.6)	134 (92.4)	1.24 (0.60–2.54)	0.57	1.25 (0.59–2.62)	0.56
Severe anemia (< 7.0 g/dL)	1 (2.7)	8 (1.3)	2.5 (0.293–20.847)	0.41	33 (8.8)	341 (91.2)	5.17 (1.24–21.62)	*0.025*	6.5 (1.49–28.16)	*0.013*

Adjusted for gravidity, ITN use, IRS use, IPTp-SP use and maternal anemia

### Prevalence of fetal anemia and associated risk factors

Out of the 631 babies, the mean fetal Hb concentration was 15.36 ± 1.87 g/dL, while 37 (5.9%) had cord Hb of less than 12.5 g/dL. Gravidity, gestational age, malaria infection at delivery and the use of IPTp-SP were not associated with fetal anemia. Also, the age of participants, marital status, education level, number of visits to the antenatal clinics, use of mebendazole, IRS, ITN, and FEFO were not associated with fetal anemia. Furthermore, maternal anemia was not a risk factor for fetal anemia even after stratifying for the level of anemia as mild, moderate and severe anemia (Table 3).

## Discussion

This study assessed the prevalence of maternal anemia, LBW, fetal anemia and preterm delivery among pregnant women at delivery in an area with a low prevalence of malaria using various doses of IPTp-SP. The overall prevalence of maternal anemia was 40.6%, indicating that the prevalence of maternal anemia at delivery is still high despite the reduced malaria transmission, reported increased uptake of FEFO supplements and other preventive measures for malaria during pregnancy. A high prevalence of anemia in these women could be due to several factors, including poor nutritional status, adherence to FEFO, mebendazole and IPTp-SP. Although FEFO and mebendazole were provided to almost all pregnant women, adherence could not be ascertained. Besides, there are reported frequent stock out of SP in the health facilities, and therefore pregnant women may be required to purchase SP from private pharmacies to use them at home [16, 17]. Therefore, if not given under directly observed therapy, adherence to the use of IPTp-SP as prescribed may not be guaranteed.

In this study, the 40.6% prevalence of maternal anemia is relatively lower than 50% that was reported in the study conducted in the same area in 2012 [14]. In 2012, malaria transmission in Dar es Salaam was 3.6% compared to 1.1% reported in this study. Therefore, the decline in malaria transmission may be one of the reasons for reduced maternal anemia in this area [12, 18]. Also, for the past few years, efforts have been made in Tanzania in ensuring that all the recommended preventive measures for malaria and anemia in risk groups are implemented [19].

Studies conducted in Northern and Eastern Tanzania between 2002 and 2004 reported the prevalence of maternal anemia of 47.4 and 68% prevalence respectively [9, 20]. The prevalence of malaria in these studies was respectively twice and six times higher than that in our study. Besides, the studies included HIV positive pregnant woman [9, 11]. Malaria and HIV independently cause maternal anemia, and the prevalence is higher in malaria-HIV co-infection [21]. Our study excluded HIV positive participants. Therefore, despite all interventional measures for the prevention of malaria and anemia in pregnancy, with a substantial reduction of malaria in this area, the prevalence of maternal anemia reported in our study is still very high. As reported elsewhere, reducing malaria transmission alone reduces moderate to mild anemia by about 25% and severe anemia by 60% [6]. Therefore, the prevalence of severe maternal anemia (1.4%) in our study may be a reflection of reduced malaria transmission in the study area.

The prevalence of LBW in our study was 6.5% which is still high considering the reduced prevalence of malaria among the study participants (0.6%) who were HIV negative. This is comparable to a 6.7% prevalence of LBW which was reported in a study that was conducted in the same catchment area in 2010–2012 [14]. However, the prevalence of LBW has slightly declined compared to 14.0% when malaria was high (6.4%) in the same catchment area [9]. Our study demonstrated a significantly low prevalence of LBW among participants aged 25 to 29 years old. Although statistically was non-significant, participants aged ≤24 (8.0%) and ≥ 35 (9.8%) years old had a high prevalence of LBW (Table 2). A study conducted in Spain on Sociodemographic factors associated with LBW reported a high prevalence of LBW among babies born by mothers who had ≤19 years and ≥ 35 years of age [22]. Extreme maternal aged groups has been reported to contribute LBW in developed and developing countries [10, 22].

During the conduct of this study, the uptake of ≥2 doses of IPTp-SP was estimated to be 66.6% compared to 48.3% that was reported in 2010–2012 in Dar es Salaam [12, 18]. Therefore, the observed difference in the prevalence of LBW between the two studies could also be due to reduced malaria transmission and increased uptake of IPTp-SP in this area [23].

Despite a few malaria cases (0.6%) in our study, yet malaria infection increased the risk of LBW similar to previous reports [24, 25]. This indicates the risk of negative birth outcomes caused by malaria during pregnancy even in areas with a very low prevalence of malaria. Malaria during pregnancy affects fetal nutrient supply and blood perfusion, which cosequently affect the normal growth of the fetus and increase the risk of delivering babies with LBW [26].

WHO recommends the use of ≥3 doses of IPTp-SP in areas with moderate to high malaria transmission [27]. Our study found that the use of ≥3 doses of IPTp-SP is still protective against LBW despite very low malaria prevalence (0.6%) in the study participants. These findings are similar to those reported in Zambian study in which pregnant women who received ≥3 doses of IPTp-SP had decreased risk of delivering babies with LBW [23]. The improved infants birth weights in pregnant women using IPTp-SP in areas with a very low prevalence of malaria could be due to the therapeutic effect of SP for both malaria and non-malaria infections. As previously reported, SP has parasitic and bacterial effects which are significant contributing factors for improved infants’ birth weights born by mothers who receive IPTp-SP for the prevention of malaria [25]. Sulphadoxine is a sulphonamide that has a variable spectrum of activities against parasites and bacteria. Previously, sulphonamides were used for the treatment of *Streptococcus pyogenes*, *Streptococcus pneumonia, Chlamydia trachomatis*, *Haemophilus influenzae*, *Gardnerella vaginalis and N. gonorrhea* [25, 28]*.* Despite the weak antibacterial activity of sulphadoxine, frequent exposure of these microbes to the later as described by increased monthly uptake of a therapeutic dose of SP (from 2nd trimester to delivery) could reduce the microbial density and possible immunological reactions responsible for adverse birth outcomes [24, 25, 29].

In this study, the prevalence of preterm delivery was 9.2% which is comparable to 6.4% reported by Kamala et al. [10] in the same study area in 2018. Preterm delivery was associated with primigravidity, which has also been reported elsewhere [30]. Also, our study demonstrated an increased risk of preterm delivery among severely anemic mothers compared to non-anemic ones. These findings are similar to those of Kidanto et al. [9] which was conducted in Dar es Salaam in 2002, reported a 4-fold increased odds of preterm delivery by the severity of maternal anemia at delivery [9, 31]. Preterm term babies are at increased risk of morbidity and mortality, and therefore strategies to prevent maternal anemia should be scaled up as part of integrated antenatal care of pregnant women [32].

In the present study, the prevalence of fetal anemia was 5.9% which is high considering the reduced malaria prevalence in the study participants. Malaria and HIV are associated with an increased risk of fetal anemia [21, 33]. Studies conducted in high malaria endemic regions including HIV positive participants reported a high prevalence of fetal anemia [21, 33]. The observed fetal anemia could be due to submicroscopic malaria parasites sequestered in the placenta which was not included in the current report. In our study, other causes of fetal anemia such as hemolytic diseases were not investigated which could have also contributed to the observed prevalence of fetal anemia. The risk of foetal anemia increases with maternal heamoglobin concentration <8g/dL, preterm delivery and high placental malaria density [34]. However, this was not observed in our current report due to small sample size and reduced burden of malaria compared to previous reports. The risk factors for foetal anemia should not be neglected in this areas as the prevalence of foetal anemia is still high despite reduced malaira burden.

### Limitations

This study did not assess the types of maternal anemia or causes of anemia such as hereditary diseases and nutrition status. Although there was evidence of prescribing and issuing of FEFO and mebendazole in pregnant women at the ANC, their actual use was self-reported because these medications are not given under directly observed therapy. Therefore, optimal adherence to these medications by all pregnant women cannot be assured. Malaria was diagnosed using the routine surveillance and passive case detection method (mRDT) instead of Malaria epidemiological survey techniques such as nucleic acid sequence-based amplification which was not available but is recommended by WHO in areas of low malaria transmission. Therefore, the prevalence of malaria reported in this study could be underestimated due to the limited sensitivity of mRDT as compared to more advanced diagnostic methods such as nucleic acid sequence-based amplification. Estimation of gestation age using the date of last menstrual period has the potential of recall bias. The collection of post-natal blood may have overestimated anemia prevalence. Studies with robust design should be conducted in low malaria transmission areas to ascertain the causes and propose the best interventions to prevent maternal anemia and LBW.

## Conclusion

Despite the reduced malaria transmission and increased uptake of malaria preventive measures in some areas like Dar es Salaam in Tanzania, the prevalence of maternal anemia, LBW, fetal anemia and preterm delivery is still high. In this study, severe maternal anemia increased the risk of delivering preterm babies. The risk of LBW decreased in women who had used ≥3 doses of IPTp-SP during pregnancy.

Further studies should be conducted to measure adherence levels of FEFO and other medications that are used for the prevention of anemia during pregnancy. Also, causes of LBW, maternal and fetal anemia should be investigated in areas with reduced prevalence of malaria to institutes appropriate interventions against preterm delivery, LBW, fetal and maternal anemia. The recommended ≥3 doses of IPTp-SP should continue to be provided even in areas with a substantial reduction of malaria. We recommend further studies to determine why IPTp-SP improves birth weight, independent of malaria.

## Data Availability

The dataset generated and/or analyzed during this study is available from the corresponding author upon reasonable request.
